# Enhanced energy transport owing to nonlinear interface interaction

**DOI:** 10.1038/srep19628

**Published:** 2016-01-20

**Authors:** Ruixia Su, Zongqiang Yuan, Jun Wang, Zhigang Zheng

**Affiliations:** 1 College of Information Science and Engineering, Huaqiao University, Xiamen 361021, China; 2 Department of Physics and the Beijing-Hong Kong-Singapore Joint Centre for Nonlinear and Complex Systems (Beijing), Beijing Normal University, Beijing 100875, China; 3 Science and Technology on Plasma Physics Laboratory, Research Center of Laser Fusion, China Academy of Engineering Physics, Mianyang 621900, China; 4 Key Laboratory of Enhanced Heat Transfer and Energy Conservation, Ministry of Education, College of Environmental and Energy Engineering, Beijing University of Technology, Beijing 100124, China

## Abstract

It is generally expected that the interface coupling leads to the suppression of thermal transport through coupled nanostructures due to the additional interface phonon-phonon scattering. However, recent experiments demonstrated that the interface van der Waals interactions can significantly enhance the thermal transfer of bonding boron nanoribbons compared to a single freestanding nanoribbon. To obtain a more in-depth understanding on the important role of the nonlinear interface coupling in the heat transports, in the present paper, we explore the effect of nonlinearity in the interface interaction on the phonon transport by studying the coupled one-dimensional (1D) Frenkel-Kontorova lattices. It is found that the thermal conductivity increases with increasing interface nonlinear intensity for weak inter-chain nonlinearity. By developing the effective phonon theory of coupled systems, we calculate the dependence of heat conductivity on interfacial nonlinearity in weak inter-chain couplings regime which is qualitatively in good agreement with the result obtained from molecular dynamics simulations. Moreover, we demonstrate that, with increasing interface nonlinear intensity, the system dimensionless nonlinearity strength is reduced, which in turn gives rise to the enhancement of thermal conductivity. Our results pave the way for manipulating the energy transport through coupled nanostructures for future emerging applications.

The thermal transport properties of nanostructured materials have attracted significant attention in recent years, because harnessing heat transfer at nanoscale is vitally important for the development of energy conversion applications, the thermal management of microelectronic and optoelectronic devices^1^,^2^,^3^,^4^,^5^,^6^,^7^,^8^,^9^,^10^,^11^,^12^,^13^,^14^,^15^. Considering that most nanostructures are supported or surrounded by environmental materials, it is necessary to elucidate interface effects on thermal transfer through the nanosctructures.

In non-metallic and semiconductor nanostructures, energy is carried predominantly by lattice vibrations or phonons. Usually, the energy transfer in these nanomaterials is dramatically affected by the scattering of phonons at interfaces that leads to a shorter phonon mean free path (less than that of the single free-standing nanostructure). As a result, the thermal conductivity of a nanostructured material assembly will be lower than that of the free-standing individual nanostructure^16^,^17^,^18^,^19^,^20^,^21^,^22^,^23^. On the other hand, recent experiments demonstrated that the thermal conductivity of a bundle of nanoribbons can be enhanced by adjusting the interlayer van der Waals interactions^24^. It is also reported that the coupling of thermal materials to substrates counterintuitively enhances the thermal conductivity through double-wall nanotubes^25^, supported graphene on 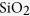 substrate^26^, double-layer graphene sheets^27^, supported silicene structures^28^ and *β*-sheet crystals of spider silk protein^29^. To get a more in-depth understanding on the important role of the interface coupling in the heat transfer through these nanostructures, the coupled nonlinear lattices, i.e., coupled Frenkel-Kontorova (FK) lattices^30^ and Fermi-Pasta-Ulam (FPU) lattices^25^,^31^, have been employed as a simplified working models of real systems. Sun *et al.* reported that the thermal transport of a FK chain-bundle can be enhanced by strong interchain Lennard-Jones (LJ) couplings^30^. However, our recent numerical results show that the energy transport in coupled chains is suppressed by strong linear inter-chain interactions^31^. Nevertheless, for strong LJ couplings, the phonon scattering arising from the nonlinear inter-chain interaction is expected to play a dominant role and suppresses the heat conduction. Thus, this common picture fails to explain the enhancement of the energy transport through couple FK chains with LJ interactions in ref. 30. These observations point to a lack of fundamental understanding on the effect of the interface nonlinearity on the heat conduction of coupled systems.

In the present paper, we focus on the energy transport in two coupled FK chains with both harmonic and anharmonic inter-chain couplings in terms of effective phonon theory (EPT) and the molecular dynamics (MD) simulations. It is found that the heat flux through the interacting FK chains increases with the nonlinear inter-chain coupling intensity for weak interface couplings. While in the case of strong nonlinear inter-chain couplings, the energy transport is found to be suppressed by the inter-chain interaction. This behavior is theoretically discussed by generalizing the effective phonon theory (EPT)^32^,^33^,^34^ to two-layer lattices and using phonon spectral energy density(SED) method^35^,^36^,^37^,^38^,^39^, and the physical mechanism of the nonlinear dependence of energy transport on the nonlinear inter-chain couplings is revealed and agrees well with numerical simulations.

## Results

### Coupled Nanostructures and FK Chains

**Figure 1:**
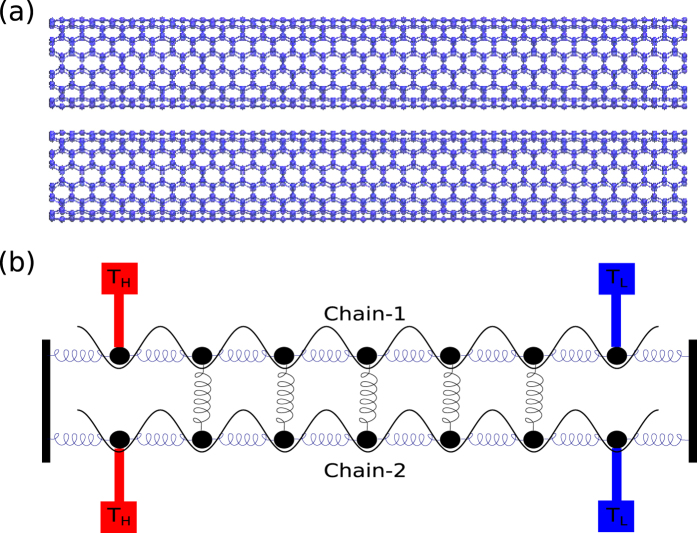
Schematics of nanotube bundles (**a**) and a coupled FK chains model (**b**).

Most nanostructures possess the assembly topology, such as double-walled carbon nanotubes, carbon nanotube bundles (see Fig. 1a), multi-layer graphene and interacting nanoribbons. These tubes, layers, or ribbons usually interact with each other through van der Waals (vdW) interactions. In order to understand the role of interface nonlinearity in heat transfer of a nanostructured material, we start with a coupled 1D FK chain model as a simplified working bench of the coupled nanostructures since the FK chains are widely used to depict the real material in condensed-matter physics and nonlinear physics^25^,^30^,^31^,^40^,^41^,^42^. The heat transfer along two identical FK chains bundled by vdW interaction is simulated to reveal the energy transport of coupled nanostructures. The total Hamiltonian can be written as


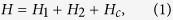



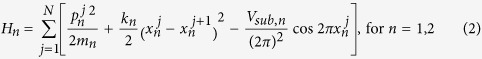



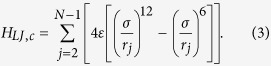


where 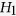 and 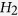 denote the Hamiltonian of chain 1 and chain 2, respectively, and 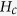 represents the interfacial coupling contribution to the total Hamiltonian. 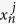 is the relative displacement of *j*-th atom in *n*-th chain, 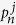 is the velocity of this particle. 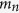, 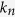 and 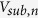 represent the mass, the harmonic coupling constant and the strength of the on-site potential of chain *n*, respectively. For simplicity, we set 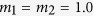, 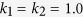 and 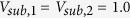. *ε*, *σ* and 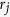 denote the intensity of the vdW interaction, the distance parameter and the distance between the *j*-th particle pair on the two sides of the vdW interface, respectively.

Following ref. 30, the dimensionless parameters used in the present paper are related to their physical quantities as follows. The vertical distance between the two chains of the vdW interface, namely, the equilibrium distance of two chains interacting with LJ potential 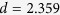, which corresponds to a real interlayer distance of graphite 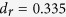 nm. The real temperature 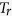 is related to the dimensionless temperature *T* by the relation 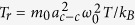. Where 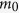 is the typical atoms mass with 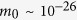 to 10^−27^ kg and 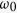 is the oscillator frequency with 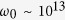 s^−1^. 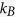 is Boltzmann constant and 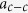 is set to be the carbon-carbon bond length 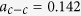 nm. The adhesion energy between two graphene sheets or collapsed CNTs is ~40 meV atom^−1^ 30,43, which corresponds to 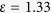 in our simulation.

To study the effect of the nonlinear inter-chain interaction, the LJ potential is expanded in a Taylor series at low temperature regime. The harmonic approximation is made by truncating the Taylor series at the second order term. Hence, by neglecting the weak nonlinear interaction, the effective harmonic potential of vdW interaction reads


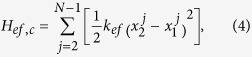


where 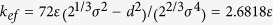 is the effective elasticity coefficient of vdW interaction.

First we perform molecular dynamics (MD) simulations to explore the difference of thermal transport between the vdW interface and effective harmonic interface. In our simulations, fixed boundary conditions are applied while the number of atoms or the size of lattice is set to be 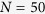. The two ends 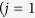 and 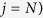 of each chain are connected to Nose-Hoover thermostats^44^,^45^ with temperatures 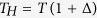 and 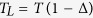, where *T* denotes the average temperature of the coupled system and Δ is the temperature difference and here 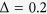. The local heat flux is defined by


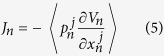


where 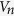 denotes the inter-particle potentail of chain *n*. Simulations are performed long enough (the total integration is 10^9^ time steps) to allow the system to reach the non-equilibrium steady state.

**Figure 2:**
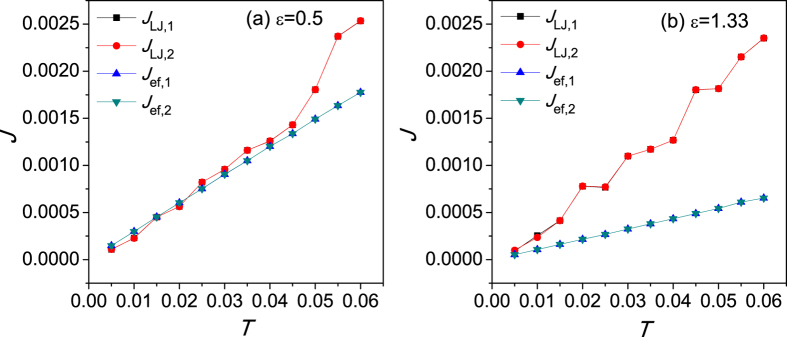
Thermal flux of the two coupled FK chains with respect to the average temperature *T* for vdW interface and effective harmonic interface for (**a**) *ε* = 0.5 and (**b**) 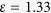. 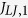 and 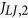 denote the heat flux of chain 1 and chain 2 with vdW interface, respectively. 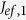 and 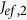 correspond to the case with effective harmonic interface.

Figure 2 shows the thermal flux of the two coupled chains with respect to the average temperature *T* for both vdW and effective harmonic interfaces. 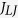, 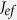 denote the heat flux of coupled system for these two cases, respectively. It is clear that in the low temperature regime, 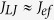, because the harmonic term in the Taylor expansion of the vdW interaction plays the dominant role when temperature is extremely low. As temperature further increases, 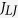 becomes gradually larger than the harmonic contribution 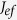. It is well known that the nonlinear contribution of the vdW interaction increases as temperature increases. Therefore, the nonlinear interaction of interface may significantly affect the thermal transport of coupled systems.

### Dependence of the Energy Transport on the Nonlinear Intensity of Inter-chain Couplings

According to Eq. (4), both the linear and nonlinear parts of the LJ potential depend on parameters *ε* and *σ*, hence it is out of the question to investigate the effect of the nonlinear contribution to the heat conduction with fixed linear couplings by varying the LJ parameters (*ε* or *σ*). We simplify the coupled model to investigate the energy transport of coupled FK chains. The simplified model has the Hamiltonian


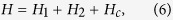



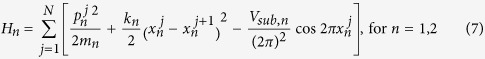



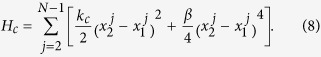


The inter-chain couplings include the harmonic interaction with strength 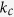 and the anharmonic interaction with strength *β*, which is the simplest model to study the effect of interface anharmonicity on heat transport. Thus, we are able to study the nonlinear effect of the inter-chain coupling by varying *β*. Particularly, if 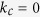, the interface is pure nonlinear. It is noted that the motion of the particles is restricted in the *x* direction, so only the inter-chain interaction in the *x* direction is considered in this model^25^,^31^. Here the thermostats temperatures of the two ends of each chain are set to be 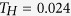 and 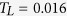.

**Figure 3:**
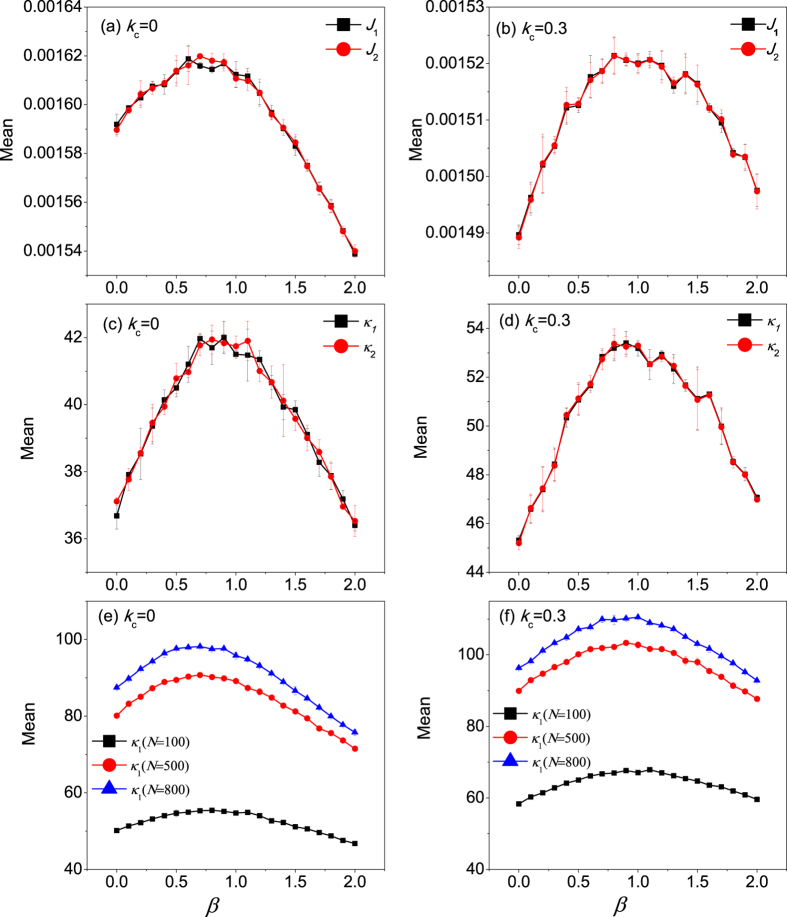
Dependence of the heat flux and thermal conductivity of coupled FK chains on the nonlinear intensity of inter-chain couplings *β* for (**a**,**c**) 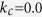; (**b**,**d**) 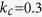; Thermal conductivity of larger scale system for (**e**) 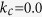 and (**f**) 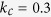. 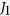 and 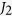 denote the heat flux of chain 1 and chain 2, respectively. 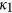 and 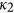 are the thermal conductivity of chain 1 and chain 2, respectively.

Figure 3(a,b) show the heat flux with variation of the interfacial nonlinearity (*β*) for 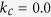 and 0.3. 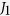 and 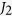 denote the heat fluxes of chains 1 and 2, respectively. For weak nonlinear intensity of inter-chain couplings (low *β* regime), the heat current increases with *β*. Thus, phonon transport through the coupled FK chains can be enhanced by the weak interfacial nonlinearity. Heat current reaches its maximum at an intermediate intensity of the interfacial nonlinearity. When *β* increases further, an inverse relationship between the heat flux and *β* is clearly exhibited, i.e., heat conduction is suppressed by the strong nonlinear interfacial coupling, which is similar to the results obtained in ref. 31. To accurately estimate the thermal conductivity of the system, we need to know the accurate temperature gradient. But there always exists a temperature jump at the boundary, so we have to obtain the temperature gradient by linearly fitting the temperature distribution after neglecting the boundary effect^40^. By combining Fig. 3(a,b) with the temperature gradient obtained from the temperature distribution of molecular dynamics simulation, the dependence of thermal conductivity on interfacial nonlinearity (*β*) is shown in Fig. 3(c,d). It is observed that the thermal conductivity through the interacting FK chains also depends nonlinearly on the inter-chain coupling nonlinearity which is similar to the interaction dependence of heat flux. The size effect on this phenomenon is shown in Fig. 3(e,f). It is found that the tendency of the nonlinear dependence of heat conductivity on interface nonlinearity is insensitive to the system size. Since chains 1 and chains 2 are identical, their numerical results are supposed to be exactly consistent with each other, so we only present the heat conductivity for chain 1 here. Moreover, the error bars are illustrated by setting varying initial conditions. It is clearly seen that the error bar is small and the phenomenon observed in our work is independent of initial conditions.

Usually, it is believed that the nonlinear effect should give rise to phonon-phonon scattering and hinder the energy transport. However, in the present paper, we find that the nonlinear inter-chain coupling counterintuitively plays a positive role in the heat conduction of coupled FK chains which validates our above speculation.

### Effective phonon theory of coupled system

To understand the underlying mechanism of the counterintuitive result obtained above, the mode-dependent thermal conductivity of the coupled FK chains should be investigated. Before quantifying the contributions of the lattice vibrations to heat transport, an exact knowledge of phonon spectra is necessary. Next we resort to the effective phonon theory (EPT)^32^,^33^,^34^,^46^,^47^. For isolated 1D anharmonic lattices, it has been verified that the effective phonon theory is a general theory to predict the phonon spectra and the heat conduction behavior. An analytic formula for heat conductivity can be derived from EPT, by which the size and temperature dependence of the heat conductivity of 1D lattice models can be analyzed. In the present paper, we propose the effective phonon theory for the coupled chains.

Consider two identical coupled 1D anharmonic (nonlinear) lattices with the general form of Hamiltonian,


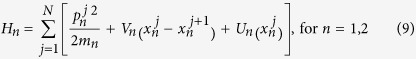



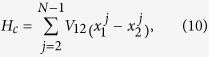


Here, for simplicity all masses are set to be 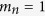 for two chains. Without loss of generality, the inter-particle potential 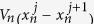, the on-site potential 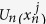, and the inter-chain interaction 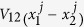 are expressed as the following infinite-order polynomials


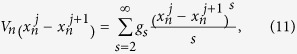



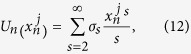



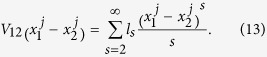


The canonical transformation which diagonalizes the harmonic Hamiltonian of chain 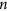 is


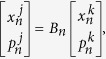


where 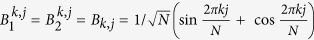 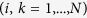. The phonon spectrum of the harmonic isolated lattice is 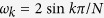, 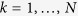. If the mode index *k* is replaced by the wave vector *k* with the relationship, 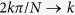, 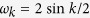. Under ergodic hypothesis, the coupled system obeys the generalized equipartition theorem,


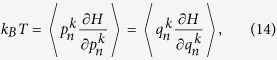


and 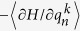 is the force in *k* space for general nonlinear lattices *n*. For coupled-lattice system, the force of each lattice in *k* space is


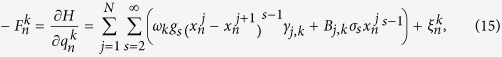


where


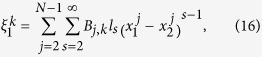



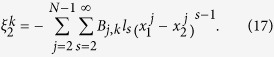


The transformation matrix 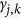 is defined as 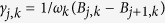 and it satisfies 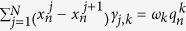.

The generalized equipartition theorem leads to


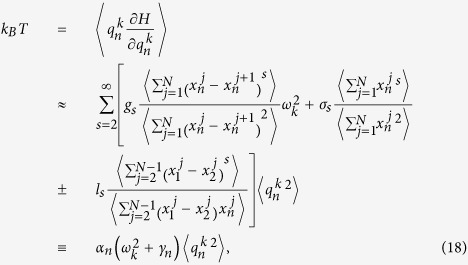


where in the third term of the above expression one takes “+” for 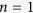 (lattice 1), and “−” for 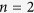 (lattice 2). The two system coefficients 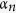 and 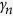 are renormalized coefficients that are defined as follows,


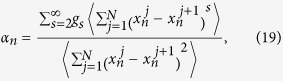



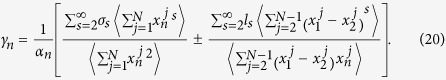


In analogy with a harmonic lattice where 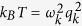, we are able to derive the expression of renormalized phonon spectrum for general 1D coupled system with and without on-site potential. It can be identified from the contribution of differences of displacements of two chains that the above renormalized dispersion relation gives the optical phonon branch


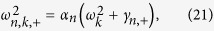


where 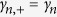. The acoustic dispersion relation for a coupled system should be equivalent to the dispersion relation of an isolated chain, which can be expressed as


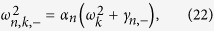


where


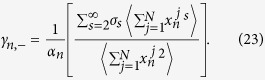


**Figure 4:**
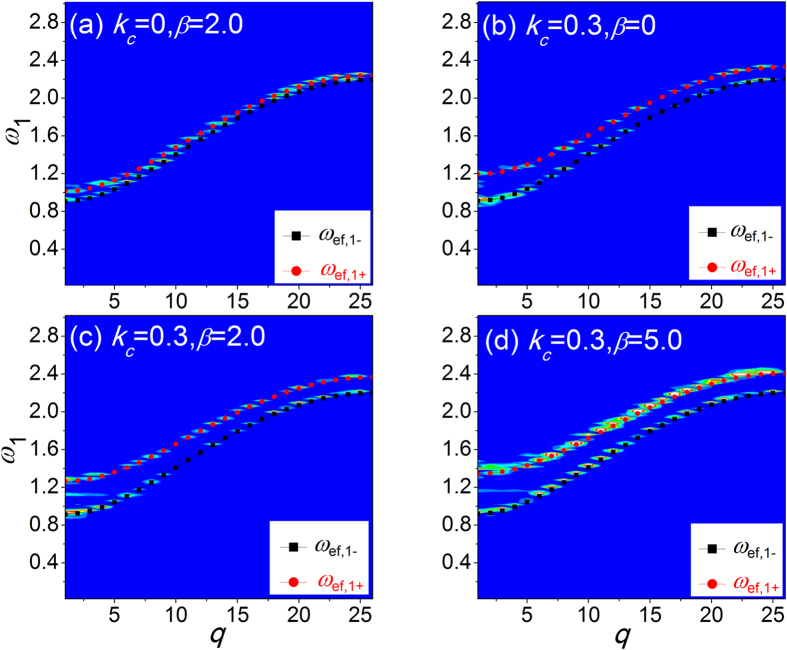
The phonon dispersion relation of chain 1 with different interfacial parameters: (**a**) 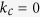, 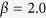; (**b**) 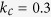, 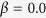; (**c**) 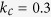, 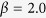; and (**c**) 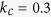, 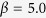. The contour plots are the results by SED method and the red and black dotted lines are results of EPT.

In Fig. 4, the dispersion relation of coupled FK chains derived from effective phonons theory is plotted by the dotted lines. It is clear that there are two phonon branches if the two chains are coupled with each other, namely, the acoustic phonon branch 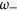 and optical phonon branch 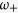. For the optical branch, the two bundled particles (with the same subscript *j*) on the two sides of the interface move in the opposite directions. For the acoustic branch, the bundled *j*-th particle pair behaves in the manner of the center of mass motion, and the acoustic dispersion relation is equivalent to the case of a single free-standing chain^31^.

Another method, i.e., the phonon spectral energy density(SED) method^35^,^36^,^37^,^38^,^39^, which is recently developed and has been used to predict the fully anharmonic phonon properties of nanostructures, is employed to verify the EPT calculations. The expression of SED is as follows


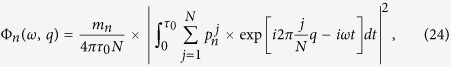


where *ω* and 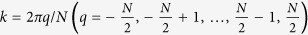 denote the phonon frequency and wavevector, respectively. *i* is the imaginary unit and 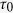 is the simulation time. Figure 4 presents the SED patterns (the contour plots) with different interfacial parameters.

It can be clearly observed that the phonon spectra by EPT are in good agreement with the predictions by SED. This verifies the emergence of the optical branch in two coupled 1D nonlinear lattices and highlights that EPT is able to predict the dispersion relation of the coupled systems quite well. Thus, we can investigate the phonon transport behavior in 1D coupled nonlinear system by EPT.

### Analysis on the mechanism of heat conductivity enhancement

For two coupled nonlinear lattices, the effective phonons can be treated as the energy carriers and the corresponding velocities are


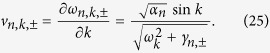


In the weak inter-chain coupling regime, we assume that the phonon scattering due to interface coupling is too weak to have a great effect on the phonon free time, and the expression of phonon lifetime is analogous to that in the isolated case and it is defined by 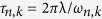, where λ depends on the lattice parameters and temperature^32^. By EPT, 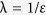, where *ε* is the dimensionless nonlinearity strength defined as the ratio between the average nonlinear potential energy and the average total potential energy which consists of both linear and nonlinear potential energy of the total system,


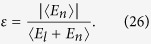


For isolated 1D anharmonic lattices, the expression of relaxation time has been verified and is an essential part of EPT^32^,^33^,^34^, by which we can qualitatively predict the heat conduction behavior. The Debye formula of thermal conductivity for an isolated chain reads


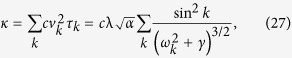


where *c* is the specific heat^48^,^49^. For coupled systems, there are two phonon branches for each chain. Thus the heat conductivity of each chain consists of two parts: the contributions of acoustic phonon branch and optical phonon branch. Therefore, the heat conductivity of the coupled system can be expressed as


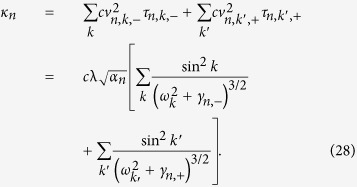


**Figure 5:**
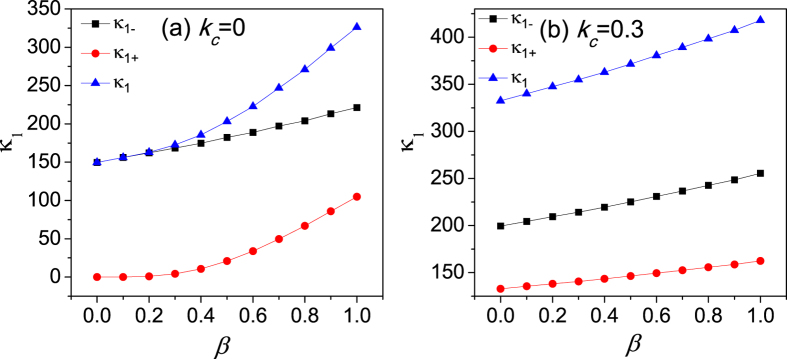
Heat conductivity of chain 1 as a function of the interface nonlinearity (*β*) by EPT for (**a**) 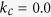; (**b**) 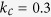.

The thermal conductivity of chain 1 as a function of the interfacial nonlinearity *β* by EPT is plotted in Fig. 5. In Fig. 5, 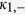 and 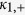 denote the contributions from the acoustic phonon branch and the optical phonon branch, respectively. 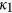 is the total thermal conductivity of chain 1. It is clearly seen that thermal conductivity increases with *β*, which gives qualitative agreements with the simulation results in Fig. 3. However, due to the rough estimation of the phonon lifetime, the calculation here is only semi-quantitive.

**Figure 6:**
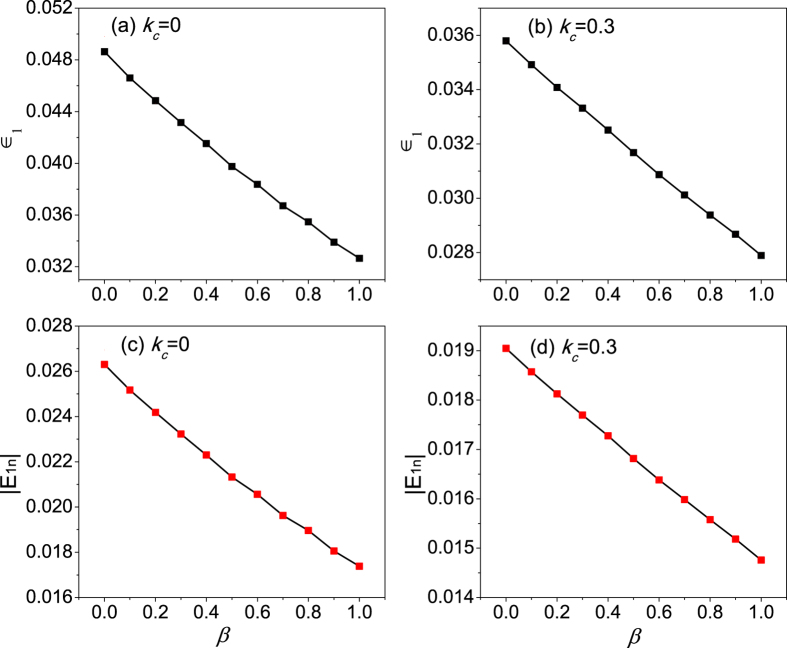
The interfacial nonlinearity dependence of dimensionless nonlinearity strength *ε* for (**a**) 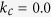 and (**b**) 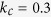. The total nonlinear energy 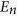 vs the interfacial nonlinearity *β* for (**c**) 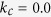 and (**d**) 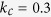.

In order to understand the enhancement of thermal conductivity, we calculate the interfacial nonlinearity dependence of the dimensionless nonlinearity strength *ε* for the whole coupled systems in Fig. 6(a,b). It is found that *ε* decreases with the increasing of *β*. Consequently, the phonon lifetime increases according to the equation 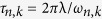 and the thermal transport is improved with increasing the nonlinear intensity of inter-chain couplings. The physical picture of the enhancement of the energy transport can be understood as follows. Although the strengthened nonlinear intensity of the inter-chain coupling gives an increasing weight to the nonlinear energy of interface, the particles are more locally confined at the bottom of the on-site potential by increasing the interface coupling. As a result, the nonlinear contribution of the intra-chain coupling decreases. Thus, the nonlinear energy of the system would not be increased by increasing *β*. On the contrary, the total nonlinear energy gradually drops as shown in Fig. 6(c,d) and *ε* decreases as shown in Fig. 6(a,b).

### Reduction of heat conduction through strongly coupled FK chains

**Figure 7:**
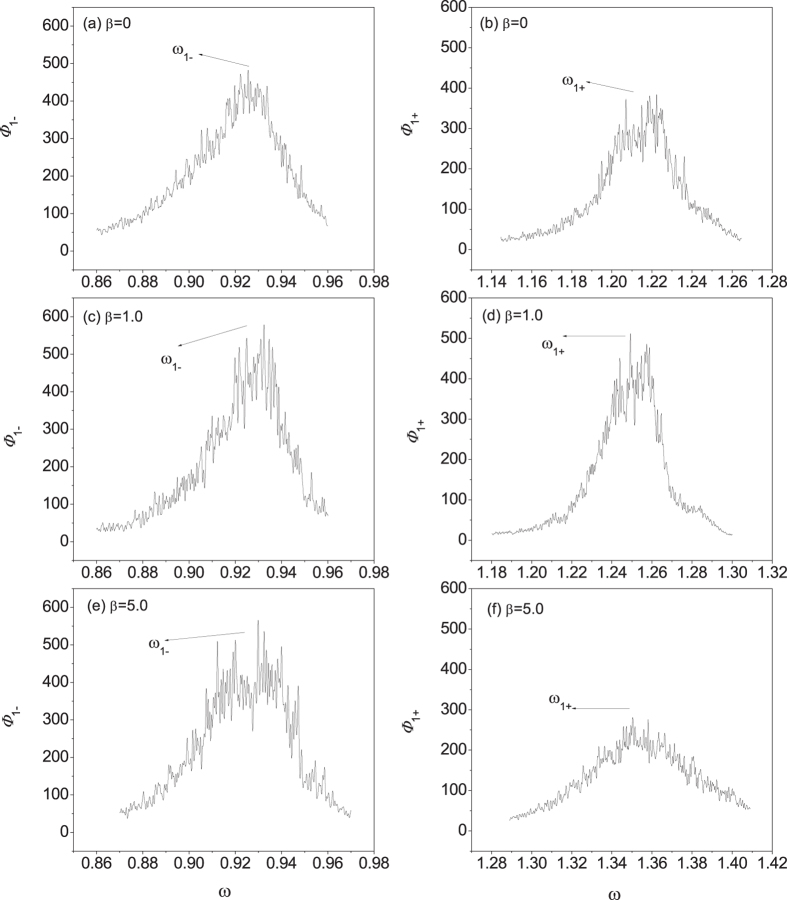
Dependence of the energy density on the frequency while *k*_*c*_ = 0.3 and *q* = 1: energy density distributions of acoustic branch for (**a**) *β* = 0.0, (**c**) *β* = 1.0, (**e**) *β* = 5.0 and energy density distributions of optical branch for (**b**) *β* = 0.0, (**d**) *β* = 1.0, (**f**) *β* = 5.0. Here 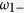, 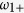 are the acoustic phonon frequencies and optical phonon frequencies of chain 1, respectively. 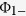, 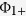 are the acoustic branch and optical branch spectral energy density of chain 1, respectively.

In the strong interfacial nonlinearity regime, the emergence of the more side peaks (messy bright lines) in the SED patterns in Fig. 4(c,d) indicates an enhanced energy exchange and a stronger phonon scattering due to the interfacial nonlinearity. To illustrate the main peaks information more distinctly, we select a narrow frequency range and show the SED distribution of the acoustic branch and optical branch versus the frequency separately with interfacial parameters (*k*_*c*_ = 0.3) when 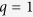 in Fig. 7. It can be clearly found that the peak gets narrower for 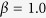 as compared with 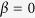, and thus this implies an increased phonon lifetime, which agrees well with the above result given by EPT. However, for large *β* 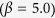 the peaks are broadened evidently, and thus the phonon lifetime is significantly reduced. As a result, the heat conduction through strongly coupled FK chains is suppressed by the strong interface phonon scattering.

### The verification of phonon lifetimes variation trend by SED

**Figure 8:**
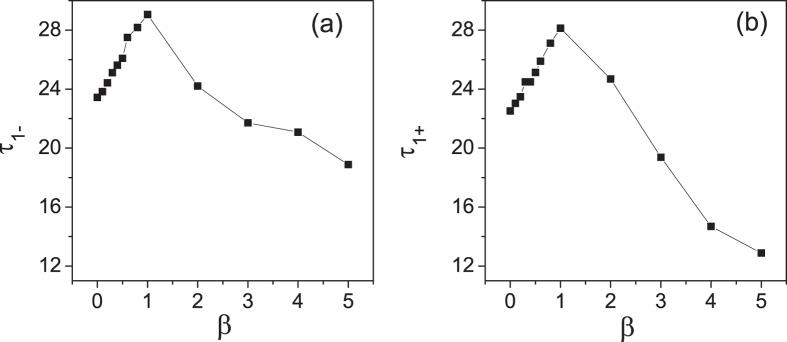
The phonon lifetimes of both the acoustic branch and optical branch versus *β* while *k*_*c*_ = 0.3 and *q* = 1 by SED. Here 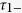, 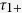 denote the acoustic phonon lifetimes and optical phonon lifetimes of chain 1, respectively.

For weak interface interactions, the enhancement of the thermal conductivity is explained by the effective phonon theory in a qualitative manner. To further verify our results, we employ the SED method to obtain the phonon lifetimes by fitting SED peaks with the Lorentzian function. In Fig. 8, the phonon lifetimes of both the acoustic and optical branches with respect to the interface parameters *β* are plotted in the case of 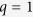. Here, only 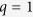 is considered because the low-*q* phonon modes contribute much to the energy transport. It is clearly observed that the phonon lifetimes rise up with increasing *β* for weak nonlinear interface interactions. Thus the prediction of phonon lifetimes from EPT is in qualitative agreement with the result obtained from SED method. If *β* is further increased, it is noticeable that the phonon lifetimes decrease after it reaches a maximum value and an inverse relationship between the phonon lifetimes and *β* is observed, which is also consistent with the result in Fig. 7.

## Discussion

In summary, we have investigated the dependence of the thermal conductivity of the coupled FK chains on the interfacial nonlinear strength *β*. It is found that thermal transport can be counterintuitively enhanced by increasing the interfacial nonlinearity. We developed the effective phonon theory of coupled system, by which the EPT calculation results are qualitatively consistent with the results by the SED calculations. Furthermore, it is found that the dimensionless nonlinearity strength *ε* of the whole couple system decreases with interface nonlinear parameter *β*, which indicates that the localization of particles leads to the enhancement of heat conductivity.

It has been reported that the thermal transport can be enhanced by increasing the strength of harmonic interface interactions in our previous work^31^. While the present paper reveals the effect of nonlinearity in the interface interaction on the phonon transport of coupled FK lattices and found that heat conduction can also be enhanced by just increasing nonlinear strength of interface. These findings indicate that it is universally applicable to enhancing thermal transport by increasing interface interactions intensity. Experimentally, we can modify the interface interaction strength by applying pressure or prestress^27^,^50^,^51^. We expect that the findings and theoretical discussions proposed in the present paper may contribute to experimental observations and shed light on manipulating the energy transport through coupled nanostructures and provides a useful guide for the thermal management of microelectronic devices and other nanostructure-based materials.

## Additional Information

**How to cite this article**: Su, R. *et al.* Enhanced energy transport owing to nonlinear interface interaction. *Sci. Rep.* **6**, 19628; doi: 10.1038/srep19628 (2016).
